# Effect of application of the thoracoabdominal rebalancing (TAR) method in moderate premature children: randomized and controlled clinical trial

**DOI:** 10.1590/1984-0462/2025/43/2024069

**Published:** 2025-01-20

**Authors:** Jaqueline Lomônaco Lemos, Denise Iunes, Aline Roberta Danaga, Carmélia Rocha, Juliana Bassalobre Carvalho Borges

**Affiliations:** a Universidade Federal de Alfenas – Alfenas (MG), Brazil.

**Keywords:** Prematurity, Thoracoabdominal, Expiratory flow, Biomechanics, Rehabilitation, Prematuridade, Toracoabdominal, Fluxo expiratório, Biomecânica, Reabilitação

## Abstract

**Objective:**

To analyze the effect of the thoracoabdominal rebalancing (TAR) method on respiratory biomechanics, respiratory discomfort, pain sensation, and physiological parameters in moderate preterm newborns, compared to a control group.

**Methods:**

This randomized clinical trial was conducted in a neonatal intensive care unit. The evaluation included: Neonatal Infant Pain Scale, physiological parameters, Silverman-Andersen score, and biomechanics (thoracic cirtometry and Charpy angle). The newborns were randomized into the TAR group (n=17) or control group (n=13) and subjected to the slow expiratory flow acceleration technique (SEFA). The evaluation of a single session was performed three times: before, after, and 30 minutes after the intervention.

**Results:**

In the intergroup comparison, there was a significant difference in respiratory rate 30 minutes after the intervention. There was no significant difference in intra- and intergroup comparisons for pain and respiratory discomfort. Regarding biomechanics, there was a significant difference in the TAR group in the Charpy angle (between assessments 1 and 2), in the axillary cirtometry (between assessments 1 and 3), and in the xiphoid process (between assessments 2 and 3). In the control group, a significant difference was observed in the axillary line (between assessments 1 and 2; 2 and 3).

**Conclusions:**

The TAR method showed a positive effect on respiratory rate and respiratory biomechanics when compared to the control group. In both groups, the techniques did not promote respiratory discomfort or pain sensation, making them safe techniques for this population.

## RESUMO

**Objetivo:**

Analisar o efeito do método reequilíbrio toracoabdominal (RTA) na biomecânica respiratória, desconforto respiratório, sensação dolorosa e parâmetros fisiológicos em recém-nascidos prematuros moderados, comparando-os com grupo controle.

**Métodos:**

Ensaio clínico randomizado realizado em unidade de terapia intensiva neonatal. A avaliação constou da Escala de Dor Infantil Neonatal, parâmetros fisiológicos, boletim de Silverman-Andersen, cirtometria torácica e ângulo de Charpy. Os recém-nascidos foram randomizados para o grupo RTA (n=17) ou para o grupo controle (n=13) e submetidos à técnica de aceleração de fluxo expiratório lento (AFEL). A avaliação de um único atendimento foi realizada em três momentos: antes, depois e 30 minutos depois da intervenção.

**Resultados:**

Na comparação intergrupos, houve diferença significativa na frequência respiratória 30 minutos após a intervenção (p=0,03). Não houve diferenças significativas nas comparações intra e intergrupos para dor e desconforto respiratório. No ângulo de Charpy, houve diferença significativa no grupo RTA, entre as avaliações 1 e 2 (p=0,01). O grupo RTA apresentou diferença significativa (p=0,03) entre as avaliações 1 e 3 para a cirtometria de linha axilar e entre as avaliações 2 e 3 no processo xifoide. No grupo controle, houve diferença significativa da linha axilar entre as avaliações 1 e 2 (p=0,04) e 2 e 3 (p<0,001).

**Conclusões:**

O método RTA apresentou efeito positivo na frequência respiratória e na biomecânica respiratória quando comparado ao grupo controle. Em ambos os grupos, as técnicas não promoveram desconforto respiratório ou sensação dolorosa, tornando-as seguras para essa população.

## INTRODUCTION

Prematurity is a complex clinical condition, being the main risk factor for morbidity and mortality in children under five years of age.^1,2^ The anatomy of the airways presents diverse characteristics, making preterm newborns (PTNB) vulnerable to respiratory complications. In this context, the role of the physiotherapist is fundamental for the development of PTNB, contributing to the reduction of morbidity and mortality, hospital stay duration, hospital costs, and improving quality of life.^3,4 5^

The physiotherapy techniques currently used are well documented in the literature, but each technique has a precise indication, considering the dysfunctional diagnosis. Thus, the physiotherapist faces the challenge of utilizing interventions that improve air flow, respiratory biomechanics, and thoracoabdominal synergy, providing greater comfort for PTNB.^6,7^

One widely used physiotherapy technique in neonatology is the increase of slow expiratory flow (SEFA), indicated for bronchial obstruction. Its use is justified by the fact that it can be applied from birth, with adaptations for each age group.^8,9^

In this sense, a method gaining attention in physiotherapy care is thoracoabdominal rebalancing (TAR), which has shown positive effects on thoracic biomechanics and reduction of respiratory muscle effort. It encourages pulmonary ventilation and promotes bronchial hygiene by reorganizing the muscular synergy between the thorax and abdomen, normalizing tone, adjusting muscle length and strength, and reestablishing the balance between inspiratory and expiratory forces.^10,11^ It involves a set of gentle maneuvers, different from conventional techniques, but it has not been sufficiently studied, especially in PTNB.^12^

There are gaps in the literature regarding safe respiratory physiotherapy techniques for this population, and studies usually focus on bronchial hygiene outcomes and adverse effects. Therefore, this study aimed to analyze the acute effect of the TAR method on respiratory biomechanics, respiratory discomfort, pain sensation, and physiological parameters in moderate preterm infants, and to compare it with a control group.

## METHOD

This is a double-blind, randomized clinical trial conducted in the Neonatal Intensive Care Unit (NICU) of a hospital in southern Minas Gerais. The study was approved by the Ethics Committee of the Federal University of Alfenas (Certificate of Presentation for Ethical Appreciation — CAAE 44647121.5.0000.5142) and registered with the Brazilian Clinical Trials Registry (RBR-2jkwm3x). Data collection took place between May 2021 and June 2022.

The inclusion criteria were moderate PTNB (born between 32 to 36.6 weeks of gestation), of both sexes, weighing up to 2500 g, admitted to the NICU, with or without mechanical ventilation, with a clinical prescription for respiratory physiotherapy, and not under analgesia and/or sedation. Excluded were those with clinical conditions contraindicating physiotherapy, congenital malformations, genetic syndromes, and those whose guardians did not agree. The gestational age was calculated using the New Ballard Method,^13^ applied by the attending physician.

Randomization was performed in two blocks of 30 participants each (TAR group — G1: TAR method, and control group — G2: SEFA technique, used as routine in the NICU), with simple randomization using the research randomizer^®^ application, by a person blind to the evaluation and therapy. The results were printed, placed in sealed brown envelopes with the PTNB’s numerical identification, and handed to the principal researcher. The envelopes were opened at the bedside at the time of application.

The environment had controlled noise and adequate lighting. The intervention occurred one hour after feeding, in the morning and afternoon. The newborns were positioned 10 minutes before the procedure in a comfortable supine position, respecting the neonatal flexor pattern, with a cushion under the scapular region, in a heated incubator, wearing only a diaper. The measuring tape for chest cirtometry evaluation was positioned in the axillary fold region to avoid manipulating the PTNB during the assessment, being subsequently moved to each cirtometry measurement level. The evaluations were conducted by the same evaluator, blind to the procedures performed, at three distinct times: evaluation 1 (before the intervention), evaluation 2 (immediately after the intervention), and evaluation 3 (30 minutes after the intervention).

The evaluator analyzed the parameters described in scales validated for the Brazilian population: the Neonatal Infant Pain Scale (NIPS),^14^ with a score above three indicating pain, and the Silverman-Andersen score (SAS),^4,15^ with a total score below five indicating mild discomfort. The heart rate, oxygen saturation, and blood pressure were recorded using the Dixtal DX 2021 multiparameter monitor, and the respiratory rate by counting for 1 minute. Respiratory biomechanics were evaluated using Charpy angle goniometry (finger goniometer, Trident^®^, with the value recorded in degrees)^16^ and chest cirtometry with a common measuring tape, considering three anatomical points: axillary fold, xiphoid appendix, and umbilical line.^17,18^

The TAR maneuvers were applied by a physiotherapist trained in the method. The SEFA maneuver was applied by another physiotherapist specialized in neonatal and pediatric intensive care, an employee of the NICU in the study who routinely uses the maneuver. Each PTNB underwent a single session (around 20 minutes), and no additional procedures were performed before or after the intervention.

For the application of the TAR method, the maneuvers included: proper positioning, stretching of inspiratory muscles, thoracoabdominal support, lower abdominal support, iliocostalis support, inspiratory assistance, and thoracoabdominal support associated with lower abdominal support. The SEFA maneuver was performed using a bimanual grip, with one hand gently compressing the anterolateral chest wall of the newborn during expiration, while the other hand provided static support on the abdomen.

The variables used for sample size calculation were inferred from the outcomes of similar studies. For the outcome of respiratory discomfort, 12 patients were required in each group. The variables had a standard deviation of 0.80. To detect a difference of 1 point and a test power of 80%, with a significance level of 5%, the data were grouped in a database (Microsoft^®^ Excel^®^ Version 2109). For statistical analysis, the R Foundation for Statistical (version 3.5.1, 64x) software was used. The data were analyzed using descriptive statistical methods, obtaining mean values, standard deviation, and confidence interval (95%CI). The data sets were then tested for normality using the Shapiro-Wilk test, and paired t-test for parametric data and Wilcoxon test for non-parametric data were performed, with a significance of 5%.

## RESULTS

**Figure 1:**
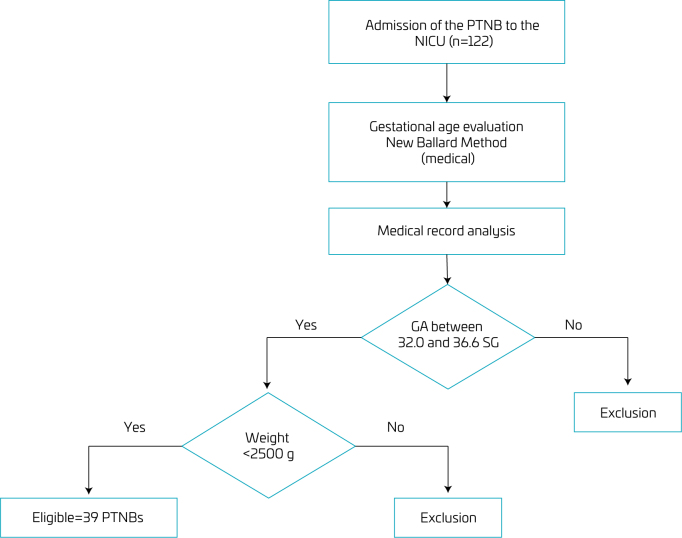
Flowchart for selecting preterm newborns

PTNBs: preterm newborns; NICU: neonatal intensive care unit; GA: gestational age; GS: gestational week.

**Figure 2:**
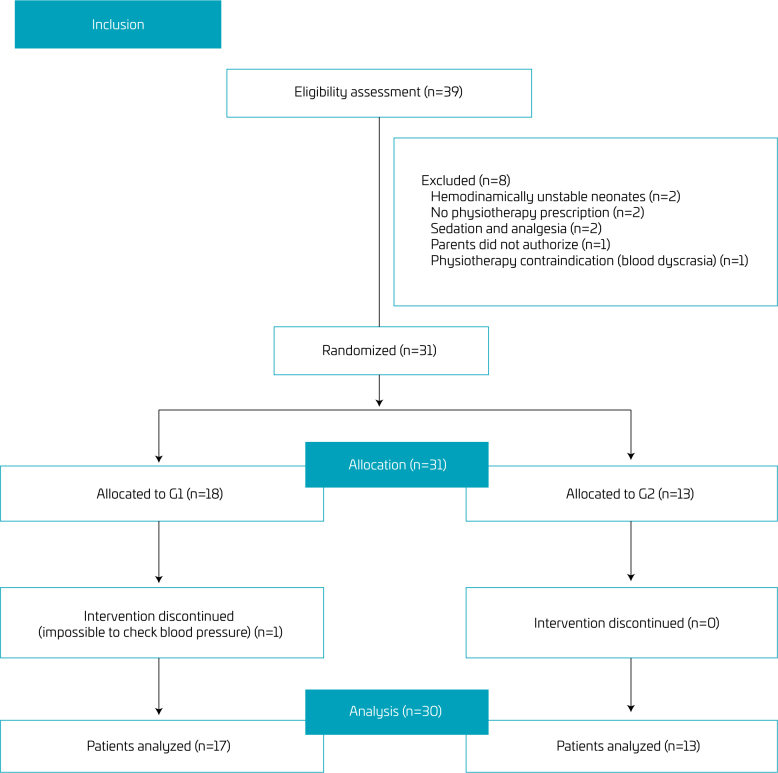
Flowchart adapted from the Consort.

TRM: Thoracoabdominal Rebalancing Method; SEFA: Slow Expiratory Flow Acceleration.

The selection of PTNB after admission to the neonatal ICU is presented in Figure 1. After analyzing the inclusion and exclusion criteria, 31 PTNBs were randomized (Figure 2).

The general characteristics of the sample are described in Table 1, showing homogeneity between the groups. The profile of the evaluated PTNBs is detailed in Table 2.

**Table 1:** General characteristics of the sample distributed between the Thoracoabdominal Rebalance Method and Slow Expiratory Flow Acceleration groups

Variables	G1–TAR (n=17)	G2–SEFA (n=13)	p-valor
GA at birth (weeks)	33.9±1.2	33.5±3.3	0.421
Corrected GA (weeks)	34.5±1.1	35.0±1.5	0.129
Days of life (collection)	4.8±1.6	5.6±1.2	0.272
Birth weight (g)	1,898±319	1,673±378	0.328
Weight on the day of application of the techniques (g)	1,789±279	1,602±307.0	0.262

GA: gestational age; TAR: Thoracoabdominal Rebalance Method; SEFA: Slow Expiratory Flow Acceleration. Data expressed in mean±standard deviations.

**Table 2:** Profile of the preterm newborns according to diagnosis and resources used in the Neonatal Intensive Care Unit.

Variables	G1–TAR (n=17)	G2–SEFA (n=13)
Sex (%)		
Female	4 (23.5)	6 (46.2)
Male	13 (76.5)	7 (53.8)
Diagnosis (%)		
RDS	14 (82.4)	8 (61.5)
ARD	2 (11.8)	2 (15.4)
Hypoglycemia	1 (5.9)	Zero
Congenital pneumonia	Zero	1 (7.7)
Venous access (%)		
No access	8 (47.1)	7 (53.8)
UVC	8 (47.1)	3 (23.1)
PVC	Zero	Zero
PICC	1 (5.9)	3 (23.1)
Phototherapy (%)	15 (88.2)	12 (92.3)
Tube (%)		
Does not use	11 (64.7)	5 (38.5)
OGT open	1 (5.9)	2 (15.4)
OGT closed	5 (29.4)	6 (46.2)
NGT closed	Zero	Zero
Ventilatory support (%)		
Room air	13 (76.5)	10 (76.9)
Macronebulization	4 (23.5)	3 (23.1)

RDS: Respiratory Distress Syndrome; ARD: Adaptive Respiratory Distress; UVC: Umbilical Venous Catheter; PVC: Peripheral Venous Cathete; PICC: Peripherally Inserted Central Catheter; OGT: Orogastric Tube; NGT: Nasogastric Tube.

The physiological parameters of respiratory rate (RR), heart rate (HR), mean arterial pressure (MAP), and oxygen saturation (SpO2) observed in the three evaluations for the TAR group (G1) were compared and showed no significant differences. The same was true for the control group (G2). Additionally, there was no significant difference between G1 and G2 in evaluations 1 and 2. However, in evaluation 3, RR showed a significant difference (Table 3).

**Table 3:** Comparison of physiological parameters, within and between groups, at the 3 assessment time points.

Variable	Groups	Mean±standard deviation		p-value	Mean±standard deviation		p-value	Mean±standard deviation		p-value
		Assessment 1	Assessment 2		Assessment 1	Assessment 3		Assessment 2	Assessment 3	
**RR**	G1	54.1±11.8	52.1±10.3	0.530	54.1±11.8	51.5±9.4	0.327	52.1±10.3	51.5±9.4	0.860
	G2	59.9±12.9	58.7±6.0	0.685	59.9±12.9	57.3±10.1	0.577	58.7±6.0	57.3±10.1	0.593
	p-value	0.181	0.143		0.181	0.030		0.143	0.030	
**HR**	G1	140.5±20.8	136.7±14.6	0.411	140.5±20.8	135.0±16.9	0.308	136.7±14.6	135.0±16.9	0.618
	G2	138.4±15.0	144.0±12.1	0.142	138.4±15.0	143.3±14.3	0.331	144.0±12.1	143.3±14.3	0.852
	p-value	0.419	0.347		0.419	0.529		0.347	0.529	
**MAP**	G1	45.3±7.5	43.4±8.7	0.509	45.3±7.5	43.5±8.2	0.525	43.4±8.7	43.4±8.2	0.985
	G2	46.6±14.3	41.7±10.5	0.334	46.6±14.3	42.8±8.7	0.472	41.7±10.5	42.8±8.7	0.383
	p-value	0.568	0.565		0.568	0.899		0.565	0.899	
**SpO2**	G1	96.7±2.9	97.4±2.5	0.192	96.7±2.9	97.2±2.5	0.301	97.4±2.5	97.2±2.5	1.000
	G2	97.3±2.2	97.2±2.1	1.000	97.3±2.2	97.0±2.8	1.000	97.2±2.2	97.0±2.8	0.959
	p-value	0.944	0.664		0.944	0.623		0.664	0.623	

G1: TAR (thoracoabdominal rebalancing); G2: SEFA (slow expiratory flow acceleration); RR: respiratory rate; HR: heart rate; MAP: mean arterial pressure; SpO_2_: peripheral oxygen saturation.

The pain intensity, assessed using the NIPS scale, showed an initial mean of less than 3 for both G1 and G2, suggesting that the newborns did not experience pain at the start of the interventions. In evaluations 2 and 3, the means remained below 3, with no significant differences observed between the three evaluation moments (Table 4).

**Table 4:** Comparison within and between groups of the analyzed variables, at the three assessed time points.

Variable	Groups	Mean±standard deviation		p-value	Mean±standard deviation		p-value	Mean±standard deviation		p-value
		Assessment 1	Assessment 2		Assessment 1	Assessment 3		Assessment 2	Assessment 3	
PAIN±NIPS)	G1	1.82±1.91	1.00±1.50	0.104	1.82±1.91	1.29±2.14	0.281	1.00±1.50	1.29±2.14	0.591
	G2	1.84±1.21	2.00±1.82	0.832	1.84±1.21	1.92 ±2.10	1.000	2.00 ±1.82	1.92±2.10	0.797
	p-value	1.000	0.160		1.000	0.354		0.160	0.354	
RD±BSA)	G1	0.4±1.0	0.4±0.6	1.000	0.4±1.0	0.5±0.7	0.860	0.4±0.6	0.5±0.7	0.860
	G2	1.1±1.2	1.2±1.5	0.772	1.1±1.2	1.2±1.3	0.765	1.2±1.5	1.2±1.3	1.000
	p-value	0.028*	0.155		0.028	0.137		0.155	0.137	
Charpy angle ±)	G1	54.5±2.5	49.8±13.2	0.006	54.5±12.5	53.3±13.1	0.349	49.8±13.2	53.3±13.1	0.012
	G2	51.2±16.4	52.8±16.3	0.384	51.2±16.4	50.7±16.0	0.707	52.8±16.3	50.7±16.0	0.064
	p-value	0.385	0.850		0.385	0.412		0.850	0.412	
Axillary line cirtometry±cm)	G1	27.1±1.5	27.6±1.7	0.149	27.1±1.5	27.6±1.7	0.027	27.6±1.7	27.6±1.7	0.746
	G2	25.7±1.9	26.6±1.9	0.036	25.7 ±1.9	27.0±1.8	<0.001	26.6±1.9	27.0±1.8	0.152
	p-value	0.081	0.213		0.081	0.589		0.213	0.589	
Cirtometry of the xiphoid process±cm)	G1	27.8±1.7	28.0±1.4	0.496	27.8 ±1.7	27.6±1.7	0.399	28.0±1.4	27.6 ±1.7	0.034
	G2	27.1±1.6	27.1±1.8	0.765	27.1±1.6	27.1±1.7	0.804	27.1±1.8	27.1±1.7	1.000
	p-value	0.740	0.354		0.740	0.701		0.354	0.701	
Umbilical line cirtometry±cm)	G1	27.4±1.5	27.2±1.6	0.275	27.4±1.5	27.1±1.8	0.266	27.2±1.6	27.1±1.8	0.626
	G2	26.5±1.8	26.4±1.8	0.337	26.5±1.8	26.5±1.6	1.000	26.4±1.8	26.5±1.6	0.460
	p-value	0.135	0.159		0.135	0.278		0.159	0.278	

G1: TAR (thoracoabdominal rebalancing); G2: SEFA (slow expiratory flow acceleration); RD: respiratory distress; NIPS: Neonatal Infant Pain Scale; BSA: Silverman-Andersen score; cm: centimeter.

Respiratory discomfort, assessed by the Silverman-Andersen score (SAS), showed no significant difference across the three evaluations for both G1 and G2, as described in Table 4. However, a significant difference was observed between G1 and G2 in the initial evaluation, though both groups had low values, indicating minimal discomfort (Table 4).

The Charpy angle for G1 showed a significant difference between evaluations 1 and 2, indicating a reduction in the angle after the TAR. There was also a significant difference between evaluations 2 and 3, suggesting that 30 minutes after the TAR method application, the Charpy angle tended to return to the initial measurement (Table 4).

Regarding thoracic cirtometry, G1 showed a significant difference in axillary line measurements between evaluations 1 and 3, and in the xiphoid process between evaluations 2 and 3, suggesting a reduction in circumference measurements after the TAR. Conversely, G2 showed an opposite response, with a significant difference only in axillary line measurements between evaluations 1 and 2, and 1 and 3, indicating an increase in thoracic diameter after SEFA application (Table 4).

Comparing the results of the NIPS scale, Silverman-Andersen score, Charpy angle, and thoracic cirtometry between G1 and G2, no significant differences were found between the variables (Table 4).

## DISCUSSION

This study analyzed the immediate and 30-minute post-application effects of the TAR method compared to a control group subjected to the SEFA technique. The results indicated that the TAR method is applicable to ICU patients, with a positive impact on respiratory biomechanics. Additionally, both the TAR method and the SEFA technique did not cause adverse effects in this sample (no increase in pain or respiratory discomfort was observed), and the TAR provided a reduction in respiratory rate (RR) as a positive impact.

Therapeutic measures directed at PTNBs aim to ensure adequate oxygenation and pulmonary ventilation while maintaining stable physiological parameters, given their deficiencies in surfactant production, structural immaturity of the airways and alveoli, thoracic alterations, respiratory muscle immaturity, and various other anatomical and physiological characteristics that influence respiratory biomechanics. Thus, physiotherapeutic intervention is necessary not only for treating respiratory diseases but also for preventing complications.^19^

Any stimulus can modify behavioral parameters and a range of physiological parameters in PTNBs, such as heart rate (HR), oxygen saturation (SpO_2_), RR, and mean arterial pressure (MAP). Studies indicate controversies about the alteration of these parameters following respiratory physiotherapy intervention, as variations may occur due to other factors such as hunger, crying, underlying pathology, or the presence of pain.^20,21,22^ In this study, no harmful effects were identified in HR, SpO2, and MAP after the intervention in both groups. Moreover, these physiological parameters remained within normal ranges 30 minutes post-intervention, indicating that the TAR method and SEFA technique do not cause instability in these patients. Regarding RR, patients subjected to TAR maintained lower values compared to the control group (SEFA), suggesting that TAR can help organize respiratory biomechanics and maintain the baby’s respiration within basal limits without excessive energy expenditure.

These findings are consistent with results from similar studies on the TAR method in analogous populations. Roussenq et al., in 2013,^6^ observed a significant decrease in RR, respiratory discomfort, and improvement in behavior in PTNBs subjected to TAR. Martins et al., in 2013,^23^ found that classical respiratory physiotherapy techniques and TAR did not trigger pain or cardiorespiratory instability in the PTNBs studied. Tassinari et al., in 2012,^24^ evaluated the influence of TAR on PTNBs’ post-respiratory distress syndrome and found no significant difference in clinical variables (HR, RR, SpO2, respiratory discomfort, and pain) pre- and post-intervention, but noted an improvement in thoracoabdominal synchrony during the method application period. Carvalho et al., in 2021,^19^ concluded that TAR had positive effects on SpO2 without affecting HR, RR, or the degree of respiratory discomfort.

The findings from the aforementioned studies align with those of the present study, where patients exhibited similar responses to the TAR method. Although no significant increase in SpO_2_ was observed post-intervention with TAR, there was no presence of pain, respiratory discomfort, or alterations in cardiorespiratory parameters post-intervention, indicating it is a safe technique for moderate preterm infants.

The concept of pain cannot be applied literally to PTNBs due to their inability to verbalize and lack of prior painful experiences that would allow for comparison and description of pain.^21^ This study showed that, for the studied population, there was no presence of pain, according to the NIPS scale, immediately after interventions in both groups. In the evaluation 30 minutes later, the patients also did not exhibit any signs of pain. Thus, it can be inferred that the proposed protocol did not cause painful effects on the patients. Several studies corroborate these findings regarding the TAR^23,24^ and SEFA techniques.^25,26^

In their study, da Silva et al., in 2022,^25^ investigated whether respiratory physiotherapy causes pain in PTNBs by comparing the SEFA technique and thoracic vibration. The NIPS scale was applied for evaluation before, during, and after the application of the chosen techniques. The patients showed no significant difference indicating pain before, during, and after technique application, nor was there a significant alteration in other observed variables (SpO2, HR, and RR). They concluded that neither technique caused pain in PTNBs as evaluated by the NIPS scale. De Moura Sousa and Nascimento Xavier, in 2013,^26^ evaluated a similar population requiring mechanical ventilation, also applying the NIPS scale at three different moments (before, during, and five minutes after the SEFA maneuver), concluding that the technique did not cause pain and that the NIPS was a useful tool to assist physiotherapy sessions, agreeing with the present study.

However, Carneiro et al., in 2016,^27^ applied the SEFA respiratory physiotherapy technique in 20 PTNBs and assessed neonatal pain using the NIPS scale before, during, and after the procedure. They concluded that the SEFA technique could trigger pain in PTNBs. This opposition may result from the technique’s application mode and the various factors that can influence the premature patient’s pain perception.

Considering respiratory discomfort, the present study’s mean SAS scores showed no differences in the two evaluations post-intervention in both groups, in intragroup comparisons. The hypothesis for this finding might be associated with the fact that most newborns had a classification of mild respiratory discomfort. Despite the initial difference, the study shows that although the difference between the groups was significant, the SEFA technique did not increase respiratory discomfort in the studied sample.

Regarding respiratory biomechanics, the present study used the Charpy angle and thoracic cirtometry as evaluation methods. The literature indicates that these evaluation methods are simple, accessible, and reliable, allowing for the identification of structural changes in the trunk’s musculoskeletal system, as well as thoracic mobility, which can directly affect pulmonary ventilation.^28^ PTNBs receiving TAR showed a reduction in the Charpy angle, favoring respiratory mechanics by improving rib positioning and the juxtaposition component, enhancing diaphragm contraction. However, 30 minutes post-intervention, the angle tended to return to its initial value, which might be justified by the fact that the proposed protocol in this study consisted of a single session. It is known that, in the clinical reality of the ICU, patients receive more sessions throughout the day, suggesting that respiratory biomechanics could be maintained with more frequent interventions. This suggests that future studies should consider multiple sessions.

This research also showed a reduction in thoracic diameter at the xiphoid process height 30 minutes post-TAR intervention, which did not occur with SEFA. Given the peculiarities of the preterm thoracic cage, such as cartilaginous and horizontal ribs, providing a more open thoracic base, this diameter reduction may suggest an adjustment in thoracoabdominal component positioning, consequently improving respiratory mechanics. In the axillary line, both TAR and SEFA showed a significant increase in diameter, indicating improved thoracic expandability and increased tidal volume during inspiration. Additionally, there was no increase in abdominal diameter post-intervention in both groups, suggesting that TAR and SEFA do not impair thoracoabdominal interaction during respiration.

Thus, considering respiratory biomechanics in its entirety, the analysis of thoracic cirtometry at the three points combined, the Charpy angle, and the reduction in RR, suggest better respiratory biomechanics organization in patients receiving TAR. This might be due to the method itself, which manipulates the thorax and abdomen, considering the “trunk” as a complex unit necessary for effective respiration.

Reinforcing some findings from this study, Ribeiro et al., in 2020,^29^ evaluated thoracoabdominal participation during respiration and its relationship with clinical risk factors in hemodynamically stable PTNBs who used oxygen or ventilatory support during hospitalization. They concluded that thoracoabdominal participation is directly related to gestational age and weight, and external factors, such as prolonged oxygen use and hospitalization duration, negatively affect the respiratory pattern. Increased abdominal participation during respiratory biomechanics may represent unnecessary energy expenditure for the patients. In this study, patients were spontaneously ventilating, with some still requiring oxygen therapy.

Limitations of the study include the difficulty in finding randomized clinical trials in the literature using the SEFA technique and/or the TAR method, and studies involving PTNB populations. Another point to elucidate is the difficulty in sample selection, as the study depends on the birth of premature babies who are stable. Given these considerations, more studies with rigorous methodology on the application of the TAR method are suggested.

The strengths of this study include the methodological rigor of a randomized clinical trial and robust data analysis. Additionally, the studied population highlights the necessity for interventional studies in PTNBs, potentially contributing to the management and choice of physiotherapeutic techniques for this population.

In conclusion, it was evident that the TAR method has a positive effect on respiratory biomechanics and respiratory frequency, when compared to the control group; its use can help in the physiotherapeutic treatment of these patients. Both the TAR method and the SEFA technique did not promote changes in other physiological parameters, respiratory discomfort and painful sensation in PTNBs, being safe techniques for this population.

## Data Availability

The database that originated the article is available with the corresponding author. CAAE: 44647121.5.0000.5142
